# MIS-C Like Features in a Patient of Atypical Kawasaki Disease: A Case Report

**DOI:** 10.31729/jnma.8538

**Published:** 2024-04-30

**Authors:** Anisha Karki, Abhishek Jha, Shova Sapkota, Nikita Kashyap, Sunil Raja Manandhar

**Affiliations:** 1 Kathmandu Medical College and Teaching Hospital, Sinamangal, Kathmandu, Nepal; 2 Department of Paediatrics, Kathmandu Medical College and Teaching Hospital, Sinamangal, Kathmandu, Nepal

**Keywords:** *aspirin*, *case reports*, *coronary vessels*, *fever*

## Abstract

Kawasaki Disease is multisystem vasculitis affecting young children and infants. While the diagnosis of a typical form of Kawasaki Disease is obvious, there are some patients who do not fulfill the classic diagnostic criteria for the disease which is termed as 'incomplete Kawasaki Disease' or 'Atypical Kawasaki Disease'. We present a case of a 6 months old child with fever who after failing to respond to IV antibiotics showed considerable improvement after administering aspirin and Intravenous Immunoglobulin thus diagnosed as Atypical Kawasaki Disease. Moreover, due to sharing of similar features by both Kawasaki Disease and Multiple Inflammatory Syndrome in Children, the case posed a diagnostic dilemma.

## INTRODUCTION

Kawasaki disease (KD) is an acute multisystem vasculitis occurring in young children.^1^ Atypical Kawasaki Disease presents with similar clinical features but doesn't fulfill the diagnostic criteria.^2^ Patient may develop coronary artery dilatation, aneurysm or myocardial infarction making it one of the leading causes of acquired heart disease in children in developed countries.^3^ Multisystem Inflammatory Syndrome in Children(MIS-C) is a rare complication temporally associated with COVID-19 typically occurring after two to six weeks of infection.^4^ Due to similar presentations of MIS-C with Kawasaki Disease, it brings a challenge in the diagnosis of the disease and therefore delay in the management.

## CASE REPORT

**Figure 1.**
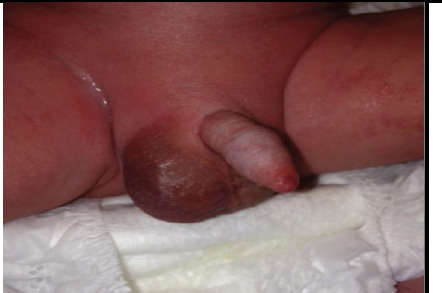
Scrotal swelling as part of generalized swelling seen in the patient.

A 6-month-old male child presented to our centre with a history of high grade continuous fever for 15 days, vomiting for 2 days and generalized swelling involving scrotum, abdomen, lower limbs (Figure 1). During the course of illness, the patient was taken to multiple hospitals where he was treated with intravenous antibiotics and other symptomatic managements for the suspicion of septic shock however the symptoms did not subside with the given treatment.

There also was a history of rashes, redness of eyes appearing on sixth day of illness, however there was no history of cough, runny nose, noisy and fast breathing, abnormal body movement, ear discharge, crying during micturition and history of aspiration of milk. His CSF culture, blood culture and urine culture done at other centres showed no growth of any microbials.

Patient was admitted to our hospital with an initial diagnosis of atypical Kawasaki disease after evaluating his symptoms and investigations. On further investigations, his hemoglobin level came out to be 8.6 g/dl and peripheral blood smear showed microcytic hypochromic anemia whereas serum iron profile revealed decreased serum iron, total iron binding capacity and increased serum ferritin level. Echo screening was done which revealed dilated coronary arteries(Left Main Coronary Artery 0.4 cm Z score 5.50; Left Anterior Descending Artery 0.3 cm Z score 6.36; Right Coronary Artery 0.2 cm Z score 1.53) but his troponin level was not raised.

The patient also showed overlapping features with MIS-C. So, SARS-COV2 (COVID-19) antibody test was done which showed it to be IgG positive. His CRP level was also increased 46.1mg/L. Also there was increased D-Dimer with elevated prothrombin time. However his total count (27,800) and platelet count (8,57,000) were high which are usually low in MIS-C. Gastric Aspirate was sent for Xpert MTB/RIF report where MTB was not detected as tuberculosis infection is still high in our country. Hence, after careful considerations a final diagnosis of atypical Kawasaki disease was made. Intravenous immunoglobulin (IVIg) 300 ml of 5 gm/ dl was transfused at the rate of 2 ml/hr for the first half hour followed by 4 ml/hr, 8 ml/hr, 16 ml/hr and 25 ml/hr respectively every half hour. Along with this, aspirin at 20 mg/kg per dose per oral was administered four times in a day; after which the patient started showing gradual improvements and was discharged after afebrile period of 48 hours with aspirin and clopidogrel. His follow up echocardiography revealed normal carotid artery diameter.

## DISCUSSION

Kawasaki Disease (KD) is an acute febrile mucocutaneous lymph node disease mainly affecting infants and young children with higher incidence in Asia and male preponderance.^1^ According to American Heart Association, KD is diagnosed in the presence of fever for at least 5 days together with at least 4 of the 5 following principal clinical features like erythema and cracking of lips, strawberry tongue, and/or erythema of oral and pharyngeal mucosa, bilateral bulbar conjunctival injection without exudate, maculopapular, diffuse erythroderma, or erythema multiforme-like rash, erythema and edema of the hands and feet in acute phase and/or periungual desquamation in subacute phase and cervical lymphadenopathy.^5^ However, patients whose illness does not meet the above KD case definition but who have fever and coronary artery abnormalities are classified as atypical KD. Significant delays in diagnosis are encountered in patients presenting with atypical Kawasaki disease which inturn increases the risk of developing coronary artery pathology.^6^ Laboratory tests typically reveal normal or elevated white blood cell count with neutrophil predominance and elevated acute phase reactants such as C-reactive protein (CRP) and erythrocyte sedimentation rate during the acute phase. Low serum sodium and albumin levels, elevated serum liver enzymes, and sterile pyuria can be present. In the second week after fever onset, thrombocytosis is common.^4,6^

MIS-C, ever since the first reported cases from UK, Spain and Italy as early as April 2020, has been studied keenly by paediatricians and rheumatologists alike, given its overlapping features with Macrophage Activation Syndrome (MAS) and Kawasaki Disease. However, distinction can be observed with MIS-C having involvement of myocardium and significant lymphopenia, neutrophilia, thrombocytopenia and elevated ferritin levels in peripheral blood. KD on the other hand has significant thrombocytosis following initial febrile stage and coronary arterial involvement in contrast to the predominant myocardial involvement of MIS-C.^7^

In addition to symptoms like fever, rash, testicular and limb swelling, our presented case had hypoalbuminemia, anemia, leukocytosis and thrombocytosis, elevated CRP and ferritin along with Z score of more than 2.5 of coronary arteries in echocardiography. Even if there were symptoms suggestive of MIS-C, the findings were not sufficient enough to make a certain diagnosis.

While patients with KD should ideally receive IVIG within 10 days of onset of fever and within 7 days to curb the coronary artery pathologies.^8^ Due to the diagnostic dilemma this case possessed, and its subsequent late presentation to our centre, this patient was started on IVIG only after 15 days of onset of disease. Despite delayed IVIG administration, the patient's symptoms improved substantially. On one month's follow up patient developed acute gastroenteritis and recovered, otherwise other clinical symptoms of the disease had subsided.
